# Chromatographic Fingerprint and Quantitative Analysis of Commercial* Pheretima aspergillum* (Guang Dilong) and Its Adulterants by UPLC-DAD

**DOI:** 10.1155/2019/4531092

**Published:** 2019-01-09

**Authors:** Jie Sun, Fang Tian, Ying Zhang, Menghua Wu, Runqian Mao, Zhiyong Le, Dongjin Xu, Hui Cao, Zhiguo Ma

**Affiliations:** ^1^College of Pharmacy, Jinan University, Guangzhou 510632, China; ^2^Research Center for Traditional Chinese Medicine of Lingnan (Southern China), Jinan University, Guangzhou 510632, China; ^3^Guangdong Province Institutes of Applied Biological Resources, Guangzhou 510260, China; ^4^Kangmei Pharmaceutical Co., Ltd., Beijing 102629, China

## Abstract

Guang Dilong is a Traditional Chinese Medicine prepared from the dried body of* Pheretima aspergillum* (E. Perrier), a species of earthworm. However, preparations of Guang Dilong may be adulterated by other species and a method of quality control is needed. A method was developed to analyze and authenticate commercial Guang Dilong, utilizing ultra-high performance liquid chromatography (UHPLC) coupled with diode array detection (DAD). Equipment included an Acquity UPLC HSS T3 column (100 mm × 2.1 mm, 1.8 *μ*m). The mobile phase consisted of acetonitrile and 0.01% formic acid, pumped at 0.3 mL/min. Wavelength detection was at 260 nm. Twenty-two batches of confirmed* P. aspergillum* samples (reference) from different sources and 20 batches of adulterated samples were analyzed to establish a reference fingerprint for commercial Guang Dilong. Five peaks in the fingerprints of the reference batches were identified as characteristic; six characteristic peaks in the fingerprints of the adulterants were identified by comparing their retention time with those of the references. The total 42 batches of samples were compared with the reference fingerprint, and the fingerprints of the* P. aspergillum* samples were similar. The UHPLC-DAD method can simultaneously determine the contents of six compounds (hypoxanthine, xanthine, uridine, inosine, guanosine, and adenosine) in the reference and adulterated batches. The six compounds showed good regression (*r* > 0.9999) within test ranges. The recovery (accuracy) was 98.25 to 101.68%, with relative standard deviation <2.67%. In summary, this UHPLC-DAD method combines chromatographic fingerprint with quantification analysis and could be readily used as an efficient quality control method for Guang Dilong.

## 1. Introduction

Guang Dilong is a Traditional Chinese Medicine (TCM) consisting of the dried body of* Pheretima aspergillum* (E. Perrier), a species of earthworm. The animal is mainly distributed in Guangdong, Guangxi, and Hainan provinces. As TCM, Guang Dilong functions as an antipyretic, antiasthmatic, and diuretic and calms the mind [1]. Modern research has also indicated that Guang Dilong exerts many pharmacological effects, such as decreasing blood pressure, preventing thrombus formation, and preventing cancer [2]. Certain preparations of Guang Dilong have been approved for clinical use in China [3].

Taxonomically, medicinal earthworms comprise three families, four genera, and 49 species [4]. Some earthworms are similar to the original animal used in Guang Dilong, but are not authentically* P. aspergillum*. Without effective methods of identification, confusion is possible. Therefore, the validation of commercial Guang Dilong is very important.

In recent years, many methods have been tried for verifying the authenticity of Guang Dilong. Such methods have included DNA (deoxyribonucleic acid) fingerprinting, microscopic character identification, qualitative identification, physicochemical identification, thin layer chromatography, gel electrophoresis, and DNA barcoding [5–11]. Thin layer chromatography and gel electrophoresis are fast and inexpensive, but results are only qualitative or semiquantitative, and therefore usually used for screening samples only. Although DNA barcodes initially seemed promising for earthworm taxonomy, species differentiation is sometimes difficult because sibling species or subspecies are possible [12]. On the other hand, ultra-high performance liquid chromatography (UHPLC) coupled with diode array detection (DAD) stands out for its high sensitivity and selectivity and its ability to perform simultaneous determinations of multiple analytes in a single run.

Guang Dilong contains nucleotides that are essential to human metabolism, such as hypoxanthine, adenine, xanthine, and guanine [13]. Nucleobases and nucleosides extracted from Guang Dilong are confirmed to have antiseizure and antiplatelet aggregation effects [14–16]. Hypoxanthine is the foundation of diastolic blood pressure, antihistamine, and asthma medicines [17]. Xanthine can expand the bronchus and has been used to treat clinical asthma [18]. Several studies have reported using UHPLC for qualitative and quantitative analyses of these components in* P. aspergillum* and other species [19–22]. However, there has been no report regarding the identification of Guang Dilong and its adulterants using nucleosides as indicators.

Combining the chromatographic fingerprint with quantitative analysis of several markers for the quality control of TCMs is an improvement over traditional methodologies. The chromatographic fingerprint in particular is important for the authenticity of herbs, while the quantification of several markers better reflects the quality of TCM [23].

The present study developed and validated a chromatographic fingerprint to differentiate Guang Dilong from its common adulterants. A new analytical method was also established to determine simultaneously the contents of six nucleoside components in Guang Dilong for evaluation of quality. This method utilizing UHPLC-DAD can be used not only for the identification of authenticity and quality evaluation of Guang Dilong, but also to guarantee the safety and effectiveness of its clinical application.

## 2. Materials and Methods

### 2.1. Chemicals and Reagents

**Figure 1:**
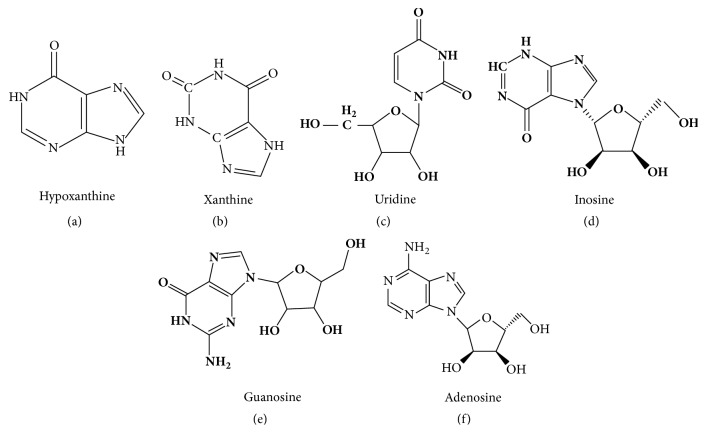
Chemical structures of the nucleoside reference components in Guang Dilong. Hypoxanthine (a), xanthine (b), uridine (c), inosine (d), guanosine (e), and adenosine (f).

The reference standards for hypoxanthine, xanthine, uridine, inosine, guanosine, and adenosine were obtained from Bio-purify Phytochemicals (Chengdu, China; Figure 1). The purity of all reference compounds was >98%, as determined by normalization of the peak areas detected by HPLC.

The Animal Genomic DNA Extraction Kit (Universal), 6× Loading Buffer (DNA), and DL 2000 DNA Marker were purchased from Tsingke (Beijing, China). Primers were synthesized in Tsingke (Beijing, China). Prime STAR Max DNA polymerase was purchased from Takara (Dalian, China). GelRed and agarose were obtained from Biotium and Aladdin, respectively (both, Shanghai, China). Acetonitrile and formic acid were of HPLC grade and purchased from Fisher Scientific (Fair Lawn, NJ, USA). All other reagent solutions of analytical grade were supplied from Sinopharm Chemical Reagent (Shanghai, China). Deionized water was obtained using a Milli-Q water purification system (Millipore, Billerica, MA, USA).

The Guang Dilong materials were purchased from China's major TCM marketplaces (Bozhou market in Anhui, Yulin market in Guangxi, and Qingping market in Guangdong). (Table 1) Dr. Zhiguo Ma authenticated all the commercial Guang Dilong samples as the dried bodies of* P. aspergillum*,* Metaphire magna, *or* Amynthas obscuritoporus* using morphological and histological methods [24]. Voucher specimens were deposited at the Research Center for TCM of Lingnan Jinan University Guangzhou, China (Southern China).

### 2.2. Polymerase Chain Reaction (PCR) Assays [9–11]

To verify the results of the morphological and histological identification, samples were identified also by DNA barcode. First, genome DNA was extracted from the samples. DNA was isolated and purified using the animal genome DNA extraction kit, in accordance with the manufacturer's instructions.

The extracted genomic DNA was used as a template for a pair of primers, which were 16Sar/16Sbr [25], 16Sar (5′-CGCCTGTTTAT-CAAAAACAT-3′)/16Sbr (5′-CCGGTCTGAACTCAGATCAC-GT -3′), amplifying the sample 16S rRNA gene through PCR. The amplification program was as follows: predegeneration, five minutes at 95°C; 40 cycles of 45 s at 95°C for degeneration, 45 s at 55.2°C for annealing, and 45 s at 72°C extensions; and the final extension was 10 min at 72°C. The PCR products were resolved via agarose gel electrophoresis, and the PCR fragment sizes were detected by DNA marker. The result of the identification was verified by DNA sequencing, whether there was a clear strip or without heteroatoms around 500 bp. Finally, the sequencing results were manually corrected and spliced and compared with the relevant 16S rRNA sequences in the GenBank database (Table 1).

### 2.3. Preparation of Standard Solutions

A mixed standard stock solution containing hypoxanthine (i), xanthine (ii), and guanosine(v) was prepared in 0.1% ammonia water, and uridine (iii), inosine (iv), and adenosine (vi) were prepared in water. The working-standard solutions were prepared by diluting the mixed standard solution with 0.1% ammonia water and water to a series of proper concentrations within the ranges: (i) 0.68-33.50 *μ*g/mL; (ii) 0.60-29.83 *μ*g/mL; (iii) 1.08-54.47 *μ*g/mL; (iv) 1.66-83.17 *μ*g/mL; (v) 1.14-56.83 *μ*g/mL; and (vi) 0.82-41.30 *μ*g/mL. All standard solutions were stored at 4°C until used and filtered through a 0.22 *μ*m membrane, prior to injection.

### 2.4. Preparation of Sample Solutions

The commercial Guang Dilong samples (1.0 g, 24 mesh) were weighed into a 100 mL conical flask with stopper, and 20 mL of 5% methanol solution was added. After soaking for 30 minutes, ultrasonication (250 W, 40 kHz) was performed at room temperature for 40 min. Then, the resultant mixture was adjusted to the original weight with extraction solvent. After centrifugation (13,000 g × 5 min), the supernatant was stored at 4°C and filtered through a 0.22 *μ*m membrane before injection into the UHPLC system for analysis.

### 2.5. Apparatus and Chromatographic Conditions

The UHPLC analysis was performed using an Agilent 1290 UHPLC system (Agilent Technologies, Waldbronn, Germany) consisting of a quaternary pump VL (G7120A), a diode array detector (G7117A, DAD), a sampler (G7129B), and a column compartment with thermostat (G7116B). The system was operated by OpenLAB CDS (ChemStation Edition) software (Agilent Technologies, Santa Clara, CA, USA). An Acquity UPLC HSS T3 column (100 mm × 2.1 mm, 1.8 *μ*m) was used and maintained at 30°C. The mobile phase was 0.01% formic acid aqueous solution (A) and acetonitrile (B). The gradient procedure was as follows: 0-1 min, 0% B; 1-1.5 min, 0-0.5% B; and 1.5-15 min, 0.5-1% B. The flow rate was kept at 0.3 mL/min. The injection volume was 3 *μ*L. The detection wavelength was at 260 nm.

### 2.6. Validation of the UHPLC Method

The calibration curve for each compound was established by plotting the peak area (*y*) versus the concentration (*x*) of each analyte. The limit of detection (LOD) and limit of quantitation (LOQ) for the six analytes were estimated at a signal-to-noise ratio of 3 and 10, respectively, by injecting a series of dilute solutions of known concentration.

The intra- and interday variations, measures of the precision of the developed method, were investigated by determining the six analytes in six replicates during a single day and by duplicating the experiments on three consecutive days. Variations of the peak area were taken as the measures of precision and expressed as percentage RSDs.

To confirm the repeatability, six solutions prepared from the same sample (No. 23) were analyzed. To confirm the stability, the sample solution (No. 23) was analyzed at 0, 1, 2, 3, and 4 hours, respectively. Variability was expressed as RSD (%).

A recovery test was used to evaluate the accuracy of this method. The recoveries of the analytes were determined by the standard addition method within the same day. A mixed standard solution was spiked into the sample (No. 23), and the recovery results were calculated as the difference between the spiked and unspiked sample analyzed under the same conditions. The recoveries were calculated according to the following formula: recovery (%) = (amount found - original amount)/amount spiked ×100%.

### 2.7. Data Analysis

Similarity analyses were performed using the following professional software, as recommended by the State Food and Drug Administration of China): Similarity Evaluation System for Chromatographic Fingerprint of TCM (Chinese Pharmacopoeia Commission, Version 2004A). The software was used to calculate the similarity between each chromatographic profile of commercial Guang Dilong samples and the simulative mean chromatogram. This approach was by way of the calculation of the correlative coefficient of the original data, based on the relative peak areas of each major constituent. Furthermore, the relative retention time and relative peak area of each characteristic peak to reference were calculated in the chromatograms.

## 3. Results and Discussion

### 3.1. PCR Identification of Combined Traits

**Figure 2:**
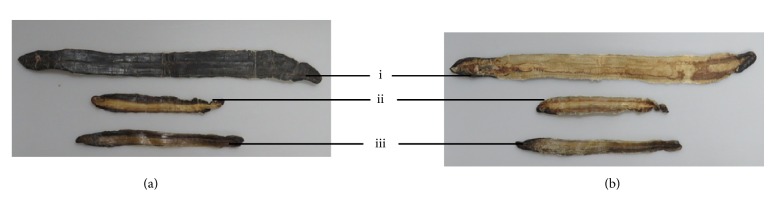
Representative samples of three species of similar earthworms. (i)* P. aspergillum*; (ii)* M. magna*; (iii)* A. obscuritoporus*; (a) outside; (b) inside.

After comparison with the GenBank database, the homology of the gene sequences for sample batches No. 1-No. 22 was above 98%, confirming that the to-be-detected tissues were* P. aspergillum* (Perrier, 1872) [26] (Table 1, Figure 2). The homology of the gene sequences for sample batches No. 33-No. 42 was above 98%, confirming that these to-be-detected tissues were* M. magna* (Chen, 1938) [26]. The results agreed with the morphological and histological identifications provided by Dr. Zhiguo Ma. However, the GenBank database does not include the gene sequence for* A. obscuritoporus *(Chen, 1938) [26]. Thus, the sample batches No. 23-No. 32 were not matched with public sequences.

### 3.2. Optimization of Extraction Procedure

To obtain satisfactory extraction efficiency, the best extraction solvent, extraction method, and extraction time were investigated. The most suitable extraction solvent was determined as 5% methanol (among 5% methanol, 10% methanol, water, and physiological saline). Ultrasonic and reflux extraction were also tested, and the former obtained better extraction.

Finally, 1.0 g of sample powder was soaked with 20 mL of 5% methanol for 20 min and then extracted by ultrasonication for 30, 40, or 50 minutes, to determine the optimal extraction time. The complete extraction of compounds could be achieved within 40 minutes. Hence, 40 minutes was chosen as the optimal extraction time.

### 3.3. Optimization of UHPLC Conditions

**Figure 3:**
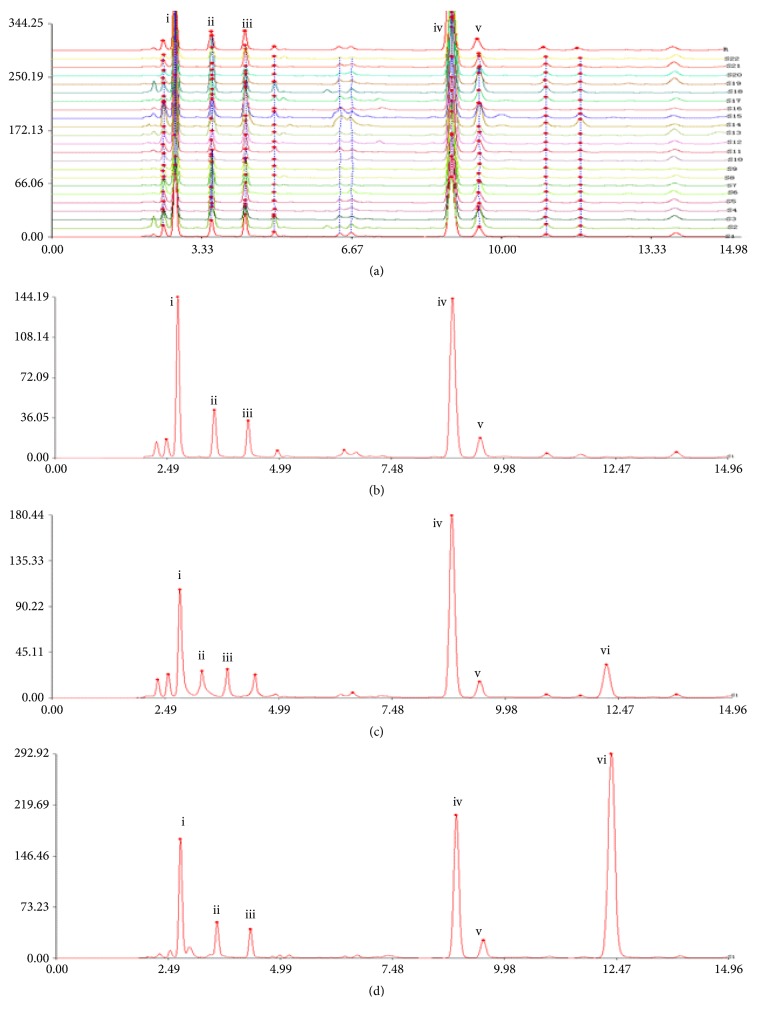
Fingerprint chromatograms of 22 batches samples of* P. aspergillum* (a);* P. aspergillum* mutual pattern (b);* M. magna *mutual pattern (c); and* A. obscuritoporus* mutual pattern (d). (i) Hypoxanthine, (ii) xanthine, (iii) uridine, (iv) inosine, (v) guanosine, and (vi) adenosine.

Different flow phases were investigated to optimize the UHPLC analysis (Figure 3). Acetonitrile was more suitable than methanol to separate the mobile phases. Formic acid was used as a mobile phase modifier to inhibit peak tailing. On the basis of the ultraviolet spectra of the six components recorded from 200 to 400 nm, 260 nm was finally selected for monitoring.

### 3.4. UHPLC Fingerprint Chromatogram Analysis

The chromatograms of the different samples had to be standardized prior to the fingerprinting analysis (Figures 3(a) and 3(b)). The process of standardization included the selection of common peaks in the chromatograms and the normalization of retention times of all common peaks. The extracts of 42 samples of Guang Dilong were selected as the sample set. Peak 4, at the retention time 9.35 minutes, was selected as the reference peak. By observing all the chromatogram peaks of* P. aspergillum*, five characteristic peaks were determined.

The same method was used to establish fingerprints for* M. magna *and* A. obscuritoporus* (Figures 3(c) and 3(d)). The fingerprints of* M. magna *and* A. obscuritoporus* each contained six characteristic peaks. Compared with the standard compounds, the six peaks were identified as hypoxanthine (i), xanthine (ii), uridine (iii), inosine (iv), guanosine (v), and adenosine (vi).

An analysis of similarity was performed for the 42 samples of commercial Guang Dilong. The similarity indexes were calculated by the mean fusion vector method. The correlation coefficients between each chromatogram of the 22* P. aspergillum* samples and the simulative mean chromatogram were as follows: 0.977, 0.997, 0.811, 0.995, 0.973, 0.948, 0.948, 0.938, 0.995, 0.998, 0.968, 0.995, 0.955, 0.997, 0.998, 0.998, 0.997, 0.879, 0.939, 0.879, 0.910, and 0.933. The similarity indexes of the 22 samples of* P. aspergillum* were higher than 0.811. This suggested that the samples from different regions shared a similar chromatographic pattern.

By comparing the common fingerprint patterns of the three earthworm species, it was found that adenosine was characteristic of the adulterants of Guang Dilong (i.e.,* M. magna *and* A. obscuritoporus*), while there was no adenosine in* P. aspergillum*. Therefore, the presence of adenosine can be used to indicate the presence of adulterants in Guang Dilong (*P. aspergillum*).

### 3.5. Method Validation and Quantitative Determination

The proposed HPLC-DAD method for quantitative analysis was validated by determining the linearity, LOD, LOQ, intra- and interday precisions, stability, and accuracy (Tables 2 and 3). All the calibration curves showed good linearity (*r* > 0.9999) within the test ranges. The overall LODs and LOQs were in the ranges of 0.0918-0.250 and 0.303-0.831 *μ*g/mL, respectively.

The RSD values of the intra- and interday variations, repeatability, and stability of the six analytes were <2.84% (Table 3). The overall recoveries lay between 98.25 and 101.68%, with RSD < 2.67%. In addition, the peak purity was investigated by analyzing the DAD data, and no indications of impurities could be found. Taken together, the results indicated that this HPLC-DAD method accurately determined six chemical markers in the samples of commercial Guang Dilong.

**Figure 4:**
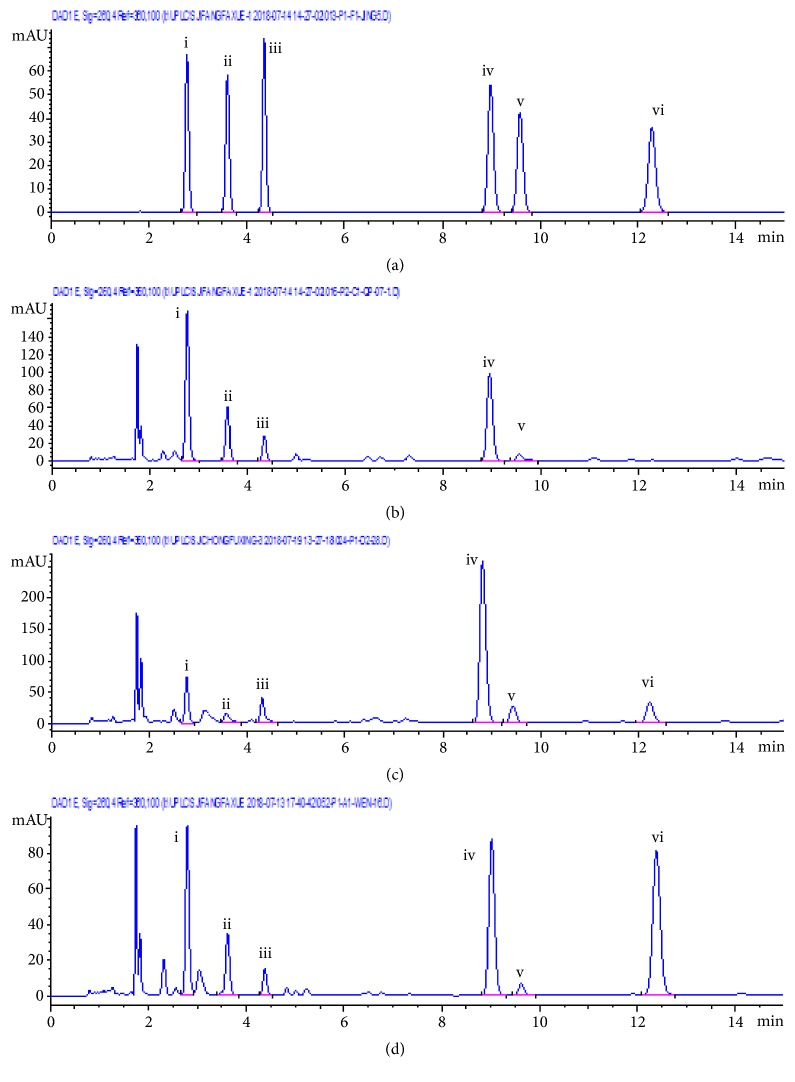
Representative UHPLC chromatograms of mixed standards (a);* P. aspergillum* (b);* M. magna* (c); and* A. obscuritoporus* (d). The peak numbers represent hypoxanthine (i), xanthine (ii), uridine (iii), inosine (iv), guanosine (v), and adenosine (vi) at wavelength 260 nm. The method involved an Acquity UPLC HSS T3 column (100 mm × 2.1 mm, 1.8 *μ*m) within 20 minutes using a gradient elution with acetonitrile/0.01% formic acid solution as the mobile phase.

The developed UHPLC method was successfully applied to simultaneously determine the following chemical markers in the 42 samples of commercial Guang Dilong (Table 4): hypoxanthine (i), xanthine (ii), uridine (iii), inosine (iv), guanosine (v), and adenosine (vi). Each sample was determined in duplicate. Peaks in the chromatograms were identified by the same retention time and on-line ultraviolet spectrum with those of the standards (Figure 4).

The content of adenosine varied greatly among the samples, from 0 to 1119.9 *μ*g/g. These differences in chemical composition may be attributable to multiple factors, such as environmental conditions and genetic variation. Thus, the establishment of a quality control method is essential to ensure product efficacy and safety.

## 4. Conclusion

For quality control of TCMs, the combination of a UHPLC fingerprint with quantitative analysis of several markers is definitely an improvement over the traditional methodologies. In the present study, a simple, accurate, and reliable UHPLC-DAD method was developed to evaluate the quality of commercial Guang Dilong. A chromatographic fingerprint for Guang Dilong was established, with simultaneous quantitative analysis of six nucleosides, namely, hypoxanthine, xanthine, uridine, inosine, guanosine, and adenosine. The method was validated as accurate and reproducible and can be readily utilized as a suitable quality control method for commercial Guang Dilong. The quantitative analysis of 42 batches of samples suggested that the presence of adenosine can differentiate adulterants from pure Guang Dilong. Therefore, the developed method can be readily used to assure the product quality of Guang Dilong and for the inspection of adulteration.

## Figures and Tables

**Table 1:** Sources of commercial Guang Dilong with PCR identification results (GenBank Accession number).

	Source	Species name	GenBank^a^
1	Guangxi	*P. aspergillum*	KF205728.1
2	Guangxi	*P. aspergillum*	KJ830749.1
3	Guangxi	*P. aspergillum*	JQ964110.1
4	Guangxi	*P. aspergillum*	JQ964110.1

5	Guangxi	*P. aspergillum*	JQ820328.1
6	Guangxi	*P. aspergillum*	JQ964109.1
7	Yulin, Guangxi	*P. aspergillum*	KJ830749.1
8	Qinzhou, Guangxi	*P. aspergillum*	KJ830749.1

9	Maoming, Guangdong	*P. aspergillum*	KJ830749.1
10	Guangxi	*P. aspergillum*	JQ964111.1
11	Guangxi	*P. aspergillum*	KJ830749.1
12	Guangxi	*P. aspergillum*	KF205728.1

13	Guangxi	*P. aspergillum*	JQ964109.1
14	Liuzhou, Guangxi	*P. aspergillum*	JQ964109.1
15	Yulin, Guangxi	*P. aspergillum*	JQ820328.1
16	Luchuan, Guangxi	*P. aspergillum*	JN187360.1

17	Luchuan, Guangxi	*P. aspergillum*	KJ830749.1
18	Luchuan, Guangxi	*P. aspergillum*	JQ964111.1
19	Luchuan, Guangxi	*P. aspergillum*	JQ964110.1
20	Luchuan, Guangxi	*P. aspergillum*	JQ964109.1

21	Yulin, Guangxi	*P. aspergillum*	JN187360.1
22	Yulin, Guangxi	*P. aspergillum*	JN187360.1
23	Guangxi	*A. obscuritoporus*	—^b^
24	Guangxi	*A. obscuritoporus*	—^b^

25	Guang Dong	*A. obscuritoporus*	—^b^
26	Maoming, Guangdong	*A. obscuritoporus*	—^b^
27	Maoming, Guangdong	*A. obscuritoporus*	—^b^
28	Hainan	*A. obscuritoporus*	—^b^

29	Rongxian, Guangxi	*A. obscuritoporus*	—^b^
30	Guangdong	*A. obscuritoporus*	—^b^
31	Guangdong	*A. obscuritoporus*	—^b^
32	Wanning, Hainan	*M. magna*	JQ904533.1

33	Qionghai, Hainan	*M. magna*	JQ904533.1
34	Guangxi	*M. magna*	JQ904533.1
35	Hainan	*M. magna*	JQ904533.1
36	Hainan	*M. magna*	JQ904533.1

37	Hainan	*M. magna*	JQ904533.1
38	Hainan	*M. magna*	JQ904533.1
39	Hainan	*M. magna*	JQ904533.1
40	Hainan	*M. magna*	JQ904533.1

41	Hainan	*M. magna*	JQ904533.1
42	Hainan	*M. magna*	JQ904533.1

^a^Accession number; ^b^not available.

**Table 2:** Regression equations, correlation coefficients, linear ranges, and LOD and LOQ of six target analytes.

	Analytes	Regression equation	*r∗*	Liner, *μ*g/mL	LOD, *μ*g/mL	LOQ, *μ*g/mL
1	Hypoxanthine	*y* = 33.148*x* – 0.7255	0.9999	0.682-33.5	0.102	0.341
2	Xanthine	*y* = 34.489*x* – 2.5796	0.9999	0.606-29.8	0.0918	0.303

3	Uridine	*y* = 22.413*x* + 0.3431	0.9999	1.08-54.5	0.164	0.542
4	Inosine	*y* = 17.695*x* + 0.5263	0.9999	1.66-83.2	0.250	0.831

5	Guanosine	*y* = 21.392*x* – 0.9505	0.9999	1.14-56.8	0.172	0.572
6	Adenosine	*y* = 31.131*x* – 0.5006	0.9999	0.824-41.3	0.125	0.412

*∗* Correlation coefficient.

**Table 3:** Precision, repeatability, stability, and recovery of six nucleosides of commercial Guang Dilong*∗*.

		Precision				
	Analytes	Intra-day	Inter-day	Repeatability	Stability	Recovery, mean (RSD%)
	Samples, n	6	3	6	6	3

1	Hypoxanthine	0.10	0.15	2.84	1.83	98.25 (2.67)
2	Xanthine	0.11	0.15	1.83	2.51	99.34 (2.57)

3	Uridine	0.10	0.19	2.00	1.65	100.69 (0.13)
4	Inosine	0.22	0.20	2.36	1.20	98.53 (1.07)

5	Guanosine	0.31	0.22	2.60	2.20	100.80 (1.67)
6	Adenosine	0.13	0.17	2.20	0.70	101.68 (0.81)

*∗*Reported as RSD%, unless indicated otherwise.

**Table 4:** Content of six components in 42 batches of commercial Guang Dilong, *μ*g/g, n = 2.

	Hypoxanthine	Xanthine	Uridine	Inosine	Guanosine	Adenosine
1	334.94	74.97	273.31	1385.52	238.03	-
2	269.84	51.37	430.44	1175.64	410.54	-
3	376.88	74.31	141.95	1366.53	173.45	-
4	151.97	38.56	155.30	1423.28	205.11	-

5	490.59	119.86	200.78	1488.43	209.50	-
6	451.66	88.54	168.29	1516.16	101.66	-
7	747.13	420.25	187.03	794.49	315.79	-
8	865.09	335.33	426.43	1847.55	378.55	-

9	651.40	71.06	213.91	1996.62	176.21	-
10	982.11	151.05	217.82	1428.58	57.85	-
11	361.08	85.21	101.72	856.37	73.14	-
12	587.68	108.87	341.48	955.73	217.59	-

13	131.05	36.01	88.29	1105.76	106.25	-
14	341.48	80.45	166.16	1140.47	184.39	-
15	652.87	206.48	427.23	1662.41	392.39	-
16	388.01	80.66	198.20	2590.25	195.38	-

17	292.67	31.51	49.08	1201.00	55.18	-
18	746.62	177.75	144.00	1510.70	132.25	-
19	457.21	89.50	95.54	1158.45	50.27	-
20	810.69	405.37	102.30	1143.91	120.23	-

21	513.11	160.13	220.05	2692.14	343.90	-
22	583.76	256.93	287.95	1932.09	203.00	-
23	503.07	82.11	143.20	991.91	84.55	819.37
24	161.59	33.43	74.94	128.67	106.76	1119.98

25	823.42	183.93	282.30	476.84	52.70	1357.74
26	377.43	137.69	145.07	1220.17	106.53	852.75
27	267.40	47.69	70.22	736.72	60.14	622.20
28	40.71	20.03	53.53	715.92	69.21	1107.87

29	300.54	69.96	70.89	302.82	48.73	303.92
30	85.15	38.27	98.16	601.35	111.02	694.25
31	138.46	72.34	115.70	1332.21	154.79	942.89
32	761.36	347.88	195.97	1103.22	187.11	128.88

33	601.76	58.22	152.63	885.40	87.35	139.22
34	332.29	64.13	131.00	1408.52	99.75	187.41
35	1018.72	328.83	151.43	191.79	860.87	135.63
36	171.51	40.68	128.51	1998.76	142.54	327.79

37	1050.56	876.82	139.77	2385.55	144.69	172.89
38	379.26	119.97	187.83	2324.06	127.85	398.14
39	150.83	18.61	97.10	1219.21	121.30	243.59
40	257.21	49.66	213.59	2029.20	168.91	197.30

41	110.27	31.60	202.07	2012.29	143.99	239.67
42	682.15	188.19	248.04	1378.25	142.54	275.75

## Data Availability

The research data generated from this study is included within the article.
